# Osteoclasts degrade bone and cartilage knee joint compartments through different resorption processes

**DOI:** 10.1186/s13075-018-1564-5

**Published:** 2018-04-10

**Authors:** Henrik Löfvall, Hannah Newbould, Morten A. Karsdal, Morten H. Dziegiel, Johan Richter, Kim Henriksen, Christian S. Thudium

**Affiliations:** 1 Nordic Bioscience, Herlev Hovedgade 205-207, 2730 Herlev, Denmark; 2 Division of Molecular Medicine and Gene Therapy, Lund Strategic Center for Stem Cell Biology, Lund, Sweden; 3 Department of Clinical Immunology, Rigshospitalet, Copenhagen University Hospital, Copenhagen, Denmark

**Keywords:** Osteoarthritis, Osteoclast, Extracellular matrix, Cartilage, Bone, Cell culture, Biomarker

## Abstract

**Background:**

Osteoclasts have been strongly implicated in osteoarthritic cartilage degradation, at least indirectly via bone resorption, and have been shown to degrade cartilage in vitro. The osteoclast resorption processes required to degrade subchondral bone and cartilage—the remodeling of which is important in the osteoarthritic disease process—have not been previously described, although cathepsin K has been indicated to participate. In this study we profile osteoclast-mediated degradation of bovine knee joint compartments in a novel in vitro model using biomarkers of extracellular matrix (ECM) degradation to assess the potential of osteoclast-derived resorption processes to degrade different knee joint compartments.

**Methods:**

Mature human osteoclasts were cultured on ECMs isolated from bovine knees—articular cartilage, cortical bone, and osteochondral junction ECM (a subchondral bone-calcified cartilage mixture)—in the presence of inhibitors: the cystein protease inhibitor E-64, the matrix metalloproteinase (MMP) inhibitor GM6001, or the vacuolar-type H^+^-ATPase (V-ATPase) inhibitor diphyllin. Biomarkers of bone (calcium and C-terminal type I collagen (CTX-I)) and cartilage (C2M) degradation were measured in the culture supernatants. Cultures without osteoclasts were used as background samples. Background-subtracted biomarker levels were normalized to the vehicle condition and were analyzed using analysis of variance with Tukey or Dunnett’s T3 post hoc test, as applicable.

**Results:**

Osteochondral CTX-I release was inhibited by E-64 (19% of vehicle, *p* = 0.0008), GM6001 (51% of vehicle, *p* = 0.013), and E-64/GM6001 combined (4% of vehicle, *p* = 0.0007)—similarly to bone CTX-I release. Diphyllin also inhibited osteochondral CTX-I release (48% of vehicle, *p* = 0.014), albeit less than on bone (4% of vehicle, *p* < 0.0001). Osteochondral C2M release was only inhibited by E-64 (49% of vehicle, *p* = 0.07) and GM6001 (14% of vehicle, *p* = 0.006), with complete abrogation when combined (0% of vehicle, *p* = 0.004). Cartilage C2M release was non-significantly inhibited by E-64 (69% of vehicle, *p* = 0.98) and was completely abrogated by GM6001 (0% of vehicle, *p* = 0.16).

**Conclusions:**

Our study supports that osteoclasts can resorb non-calcified and calcified cartilage independently of acidification. We demonstrated both MMP-mediated and cysteine protease-mediated resorption of calcified cartilage. Osteoclast functionality was highly dependent on the resorbed substrate, as different ECMs required different osteoclast processes for degradation. Our novel culture system has potential to facilitate drug and biomarker development aimed at rheumatic diseases, e.g. osteoarthritis, where pathological osteoclast processes in specific joint compartments may contribute to the disease process.

**Electronic supplementary material:**

The online version of this article (10.1186/s13075-018-1564-5) contains supplementary material, which is available to authorized users.

## Background

Osteoclasts are bone-resorbing multinucleated cells derived from hematopoietic stem cells via the monocytic lineage upon stimulation with the cytokines macrophage colony-stimulating factor (M-CSF) and receptor activator of nuclear factor kappa-B ligand (RANKL) [1]. Bone is resorbed by osteoclasts through secretion of hydrochloric acid dissolving the inorganic matrix [2] and proteases, mainly the cysteine protease cathepsin K [3–5] and matrix metalloproteinases (MMPs) [6–8], degrading the organic matrix. In addition to their well-established role in normal bone turnover, osteoclasts play important roles in diseases with progressive joint destruction, in particular in bone erosion in diarthrodial joints in rheumatoid arthritis (RA) [9–13]. Their role in cartilage and subchondral bone alterations in osteoarthritis (OA) is poorly understood [14].

The osteochondral junction is a key compartment of the joint. Consisting of non-calcified articular cartilage, calcified cartilage and subchondral bone, the osteochondral junction transforms shear stress from loading and motion into compressive and tensile stress via undulations in the extracellular matrices (ECMs) [14, 15]. Subchondral bone sclerosis and changes in subchondral bone metabolism, possibly mediated by osteoclast-osteoblast coupling [16–18], are often amongst the first detectable OA alterations [19]. In conjunction, increased osteoclast activity and subchondral bone remodeling are involved in OA development and progression [15]. Suppressing bone resorption has resulted in beneficial secondary effects on cartilage health in several pre-clinical OA studies and increased resorption has resulted in cartilage deterioration [20], demonstrating how osteoclasts can contribute indirectly to cartilage health. The differentiation, activity and survival of osteoclasts is tightly regulated in healthy individuals [21] whereas monocytes isolated from patients with OA exhibit increased osteoclastogenesis, elevated resorption, and reduced osteoclast apoptosis [22].

The idea of osteoclasts being directly involved in OA cartilage degradation is gaining increased attention. Human osteoclasts can degrade cartilage [23] and equine osteoclasts are recruited to the subchondral bone in spontaneous post-traumatic OA [24]. Furthermore, bone marrow lesions have been shown to be associated with the subchondral bone areas underlying articular cartilage lesions, suggesting osteoclast-associated cartilage-bone crosstalk [25, 26]. Resorption pits reaching from subchondral bone into articular cartilage have been described in OA [27], but which osteoclast-derived resorption processes that are involved in cartilage degradation have not been investigated. Different osteoclast substrates affect the possible ECM degradation products and the phenotype of the resorbing osteoclasts [28]. This suggests that the ECM can be a determinant of osteoclast functionality, and hence there is a need for increased understanding of osteoclast effects on other ECMs of relevance to joint pathology. MMPs [6, 8, 29, 30] and cathepsin K [3, 6, 8, 31] have long been thought to degrade both bone and cartilage, but in addition to osteoclasts there are many other potential contributors to MMP [32–34] and cathepsin K [34, 35] levels in the OA joint. Therefore, the contribution of proteases specifically derived from osteoclasts to degrading these ECMs requires further investigation.

To this date direct osteoclast-derived effects and the osteoclast processes involved in degrading the articular cartilage and the osteochondral junction compartments have not been determined. To shed light on if and how osteoclasts play a role in the degradation of the different joint components, in this study we investigated matrix-dependent and enzyme-dependent degradation processes by human osteoclasts on bovine joint tissues. To this end we exploited a novel cell culture model of osteoclast-derived bovine knee joint degradation, using osteochondral ECM (a subchondral bone-calcified cartilage mixture), articular cartilage, and cortical bone as osteoclast substrates. By measuring biomarkers of ECM turnover, we investigated the effects of osteoclast-derived resorption processes in degrading the ECMs of knee joint compartments in vitro.

## Methods

### Isolation of bovine knee joint ECMs

Three intact bovine knees, derived from two cows, were obtained from a local butcher. Articular cartilage was isolated from the femoral condyles, using a biopsy punch and a scalpel, and was immersed in liquid nitrogen to render the cartilage metabolically inactive and prevent endogenous protease activity. The cartilage slices were then stored in 70% ethanol at 4 °C until use.

The femoral condyles were then cut sagittally into slices using a Proxxon FET circular saw with a diamond-coated blade (Proxxon, Föhren, Germany). The slices were fixated in 70% ethanol for 10 min. The remaining articular cartilage was removed with a scalpel to leave mainly subchondral bone and calcified cartilage, the cartilage between the subchondral bone and the tidemark [36], in our osteochondral ECM. Using a chisel and mallet, strips of osteochondral matrix were isolated from the proximal surface of the denuded slices down to 1 mm beneath the surface. The resulting matrix strips were immersed in liquid nitrogen and crushed into small pieces using a tissue pulverizer, thereby homogenizing the ECM and allowing for even coverage of cell culture wells, to generate the osteochondral ECM used for culture. The osteochondral ECM was then stored in 70% ethanol at 4 °C until use.

Bovine femurs were fixated in 70% ethanol for several weeks after which cortical bone biopsies were drilled from the diaphyses. The resulting bone cylinders were cut into 70-μm-thick slices using a Minitom with a diamond cut-off wheel (Struers, Ballerup, Denmark). The bone slices were then stored in 70% ethanol at 4 °C until use.

### Histology

Femoral condyle slices, from before and after cartilage removal, were fixated in 4% formaldehyde and decalcified in 15% EDTA. The decalcified slices were infiltrated with paraffin using a Tissue-Tek VIP 5 Jr. (Sakura Finetek, Alphen aan den Rijn, The Netherlands), embedded in paraffin, and cut into 5–6-μm-thick sagittal sections using a HM 360 microtome (Microm International GmbH, Walldorf, Germany). The sections were stained with safranin O-fast green. Digital micrographs were obtained with an Olympus DP71 digital camera mounted on a BX-60 microscope with a 4X objective using the Olympus cellSens software (Olympus, Center Valley, PA, USA).

### von Kossa staining

Osteochondral matrix strips were stained with von Kossa staining to verify the presence of calcified ECM. Cortical bone slices were used as positive controls of calcification and decalcified osteochondral strips and cortical bone slices were used as negative controls. The tissues were washed in type I ultrapure water and then stained by incubating for 5 min in 1% silver nitrate under a lamp, 2 min in 1% pyrogallol, and 5 min in 1% sodium thiosulfate, with washes between each step. Images were obtained using a digital camera.

### Resorption assays

The resorption experiments were based on three independent resorption assays, including a small pilot (*n* = 3 per condition) and two larger trials (*n* = 6 per condition for each). Each trial used ECMs from one unique bovine knee and osteoclasts from one unique set of blood donors. Data from the different trials are shown individually—the pilot in Additional files 1, 2 and 3: Figures S1–S3 and the two larger trials in Additional files 4, 5 and 6: Figures S4–S6 and Figs. 2, 3 and 4, respectively—due to minor modifications in experimental setup, such as culture time, ECM and osteoclast origin, replicate numbers, and the number of conditions.

Prior to plating the ECMs, the osteochondral ECM was washed three times by centrifugation and medium replacement, whereas the slices of cortical bone and articular cartilage were washed with medium replacement only. Culture wells on 96-well culture plates were filled with 50 μl of medium followed by the addition of ECMs; osteochondral ECM was added with a spatula, until the bottoms of the wells were covered, and cortical bone or articular cartilage slices were added using forceps. Mature human osteoclasts derived from CD14^+^ monocytes were generated as previously described [37, 38] from the peripheral blood of anonymized blood donors obtained from a blood bank. The mature osteoclasts were lifted using a trypsin/EDTA mixture and a cell scraper, counted and seeded by adding 50 μl/well of cell suspension resulting in final culture densities of 1.0 × 10^5^ cells/well on articular cartilage and on osteochondral ECM and 5.0 × 10^4^ cells/well on the cortical bone. Two matrix-containing wells without osteoclasts were cultured in parallel on each plate to serve as background samples for biomarker analyses, these samples were analyzed in the same manner as all other samples. The plates were incubated for 1 h at 37 °C and 5% CO_2_ after which 150 μl of medium supplemented with various resorption inhibitors was added. The following inhibitors and final concentrations were selected based on previous research [39]: 300 nM diphyllin (V-ATPase inhibitor), 5 μM E-64 (cysteine protease inhibitor), 10 μM GM6001 (broad-spectrum MMP inhibitor), 5 μM E-64 and 10 μM GM6001 combined (E-64/GM6001), or dimethyl sulfoxide (DMSO) vehicle (1:2000 in medium), all of the above from Sigma-Aldrich (St. Louis, MO, USA). After 24 h the medium was changed by demi-depletion, replacing 150 μl of used medium with 150 μl of fresh medium supplemented with inhibitors, followed by another 3–4 days of culture. At the end of the culture 200 μl of medium was collected from each well and stored at − 20 °C until analysis. A graphical overview of the experimental model design can be found in Fig. 5.

### Cell culture viability analysis

After collecting the cell culture supernatants, 200 μl of medium supplemented with inhibitors and 12.5% alamarBlue (Thermo Fisher Scientific, Waltham, MA, USA) was added to the wells, resulting in a final alamarBlue concentration of 10%, and the cells were incubated at 37 °C and 5% CO_2_ for approximately 3 h. The relative viability of the different culture conditions was then assessed by measuring the alamarBlue fluorescence with excitation at 540 nm and emission at 590 nm using a SpectraMax M5 plate reader (Molecular Devices, Sunnyvale, CA, USA). Medim from wells containing only matrix and medium were used as background samples (data not shown).

### TRAP activity analysis

Tartrate-resistant acid phosphatase (TRAP) activity was assessed as a measurement of relative osteoclast numbers in the cell cultures. 2.5 μl of media from each cell culture well was added to a 96-well plate and mixed with 17.5 μl of type I ultrapure water. The samples were incubated with 80 μl of freshly made reaction buffer (0.25 M acetic acid, 0.125% Triton X-100, 0.25 M NaCl, 2.5 mM EDTA, 1.1 mg/ml of ascorbic acid, 5.75 mg/ml of disodium tartrate, 2.25 mg/ml of 4-nitrophenylphosphate, pH 5.5). The reaction mixture was incubated at 37 °C for 1 h in the dark and was stopped by adding 100 μl of 0.3 M NaOH. Absorbance was measured at 405 nm with 650 nm as a reference using a SpectraMax M5 plate reader (Molecular Devices, Sunnyvale, CA). Media from wells containing only matrix and medium were used as background samples (Additional file 7: Figure S7).

### Biomarker measurements

Calcium release was analyzed by measuring the concentration of total calcium (Ca^2+^) in medium after resorption using a colorimetric calcium assay, based on the *o*-cresolphthalein complexone (CPC) method, on an ADVIA 1800 Clinical Chemistry System (both from Siemens Healthineers, Erlangen, Germany). C-terminal type I collagen fragments (CTX-I) released from resorbed bone were measured using the CrossLaps for Culture ELISA (IDS, The Boldons, UK), which was used according to the manufacturer’s instructions. C2M, a metabolite of MMP-mediated collagen type II degradation, was measured as previously described [40]. Biomarker levels below the detection limits of the respective assays were assigned the detection limit as their value. Medium from wells containing only matrix and medium were used as background samples in all biomarker measurements (Additional file 7: Figure S7).

### Statistical analysis

For statistical analysis, the measured background samples from matrix without osteoclasts were subtracted from all osteoclast-containing samples. The background-subtracted data were normalized to the mean of the vehicle condition on the respective ECM and are presented as percentages of the vehicle. All graphs except Additional file 7: Figure S7 contain background-subtracted and vehicle-normalized data. The raw data on osteoclast-derived biomarkers levels compared to background samples (Additional file 7: Figure S7) were not statistically analyzed. All statistical comparisons were made within the same cell culture trial using all the conditions’ technical replicates. These comparisons were performed using IBM SPSS Statistics v24.0.0.0 64-bit edition for Windows (IBM, Armonk, NY, USA). The data were analyzed with one-way analysis of variance (ANOVA), with the assumption that the data were normally distributed, followed by either Tukey or Dunnett’s T3 (DT3) post hoc test depending on whether or not equal variances could be assumed based on Levene’s test. The post hoc test used for each test is specified in every graph that was statistically analyzed and when *p* values are given in the “Results” section (presented as *p*_Tukey_ or *p*_DT3_). Statistical significance was considered to be *p* < 0.05. Significance levels are reported with symbols in the figures (^*^comparisons to vehicle and ^#^comparisons to E-64/GM6001, the latter only being shown for E-64 and GM6001 to assess synergistic effects), where ^*^p < 0.05, ^**^p < 0.01, ^***^p < 0.001, ^****^p < 0.0001 and ^#^*p* < 0.05, ^##^*p* < 0.01, ^###^*p* < 0.001, ^####^*p* < 0.0001. Graphs were plotted in GraphPad Prism v7.01 for Windows (GraphPad Software, La Jolla, CA, USA) and represent group means and their respective standard error of the mean (SEM); significance levels were inserted manually.

## Results

### Osteochondral ECM characterization and model optimization

**Fig. 1:**
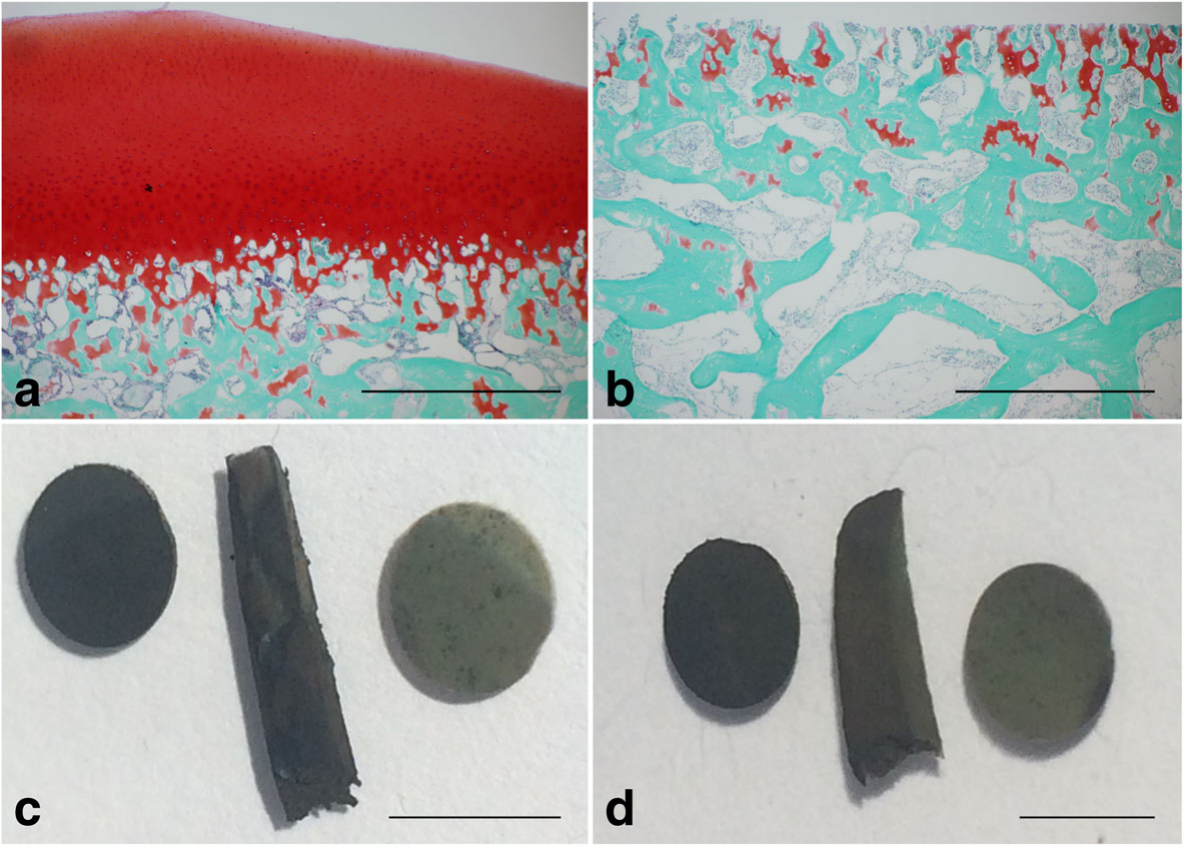
Characterization of osteochondral extracellular matrix (ECM). Femoral condyles used to isolate the subchondral matrix were stained with safranin O-fast green to verify the removal of the articular cartilage and the presence of calcified cartilage interspersed with the subchondral bone (**a**–**b**). The digital micrographs represent the status of the cartilage before (**a**) and after articular cartilage removal (**b**). Isolated osteochondral ECM strips were stained for the presence of mineralized ECM using von Kossa staining (**c**–**d**). The osteochondral strip in **c** (center) was un-treated whereas the osteochondral strip in **d** (center) was decalcified prior to staining. Both strips are displayed next to cortical bone slices (**c**–**d**, left) and decalcified cortical bone slices (**c**–**d**, right), serving as references of calcified matrix. Scale bars represent 1 mm (**a**–**b**) and 6 mm (**c**–**d**)

The osteochondral ECM was characterized by assessing the presence of calcified cartilage interspersed with subchondral bone, by safranin O-fast green staining of femoral condyle slices, and the overall calcification of the osteochondral matrix, by von Kossa staining of non-crushed strips of osteochondral matrix. Prior to removing the remaining articular cartilage from the partially denuded femoral condyles, the subchondral bone was still covered by some articular cartilage (Fig. 1a, stained red). No detectable articular cartilage remained above the subchondral bone plate after denuding, but calcified cartilage was still present interspersed with the subchondral bone (Fig. 1b, stained red). The amount of bone and cartilage in the slices varied throughout the matrix but we do not know to what extent. The von Kossa staining verifies the calcification of the osteochondral matrix (Fig. 1c, center), as can be seen by comparing this matrix strip with a decalcified matrix strip (Fig. 1d, center), cortical bone slices (Fig. 1c–d, left), and decalcified cortical bone (Fig. 1c–d, right) slices. The calcified osteochondral matrix was intermittently covered by a thin layer of cartilage that did not stain black (Fig. 1c, center), which was likely non-calcified articular cartilage.

To verify that osteoclast-derived biomarker release could be generated from all ECMs, background samples containing only medium and ECM was measured and compared to medium from the osteoclast-containing vehicle condition. The data clearly show that the addition of osteoclasts resulted in increased biomarker levels, with the exception of articular cartilage Ca^2+^, CTX-I, and cortical bone C2M, compared to background levels (Additional file 7: Figure S7).

To test if the crushing procedure would affect osteoclast-derived biomarker results, both cortical bone and articular cartilage were metabolically inactivated, crushed in the same manner as osteochondral ECM, and used in resorption assays. The overall effects of the different resorption inhibitors on osteoclast-derived biomarker release from crushed ECMs did not differ from sliced ECMs (data not shown) and only slices of cortical bone and articular cartilage were used in the optimized model.

### Osteoclast effects on the cortical bone compartment

**Fig. 2:**
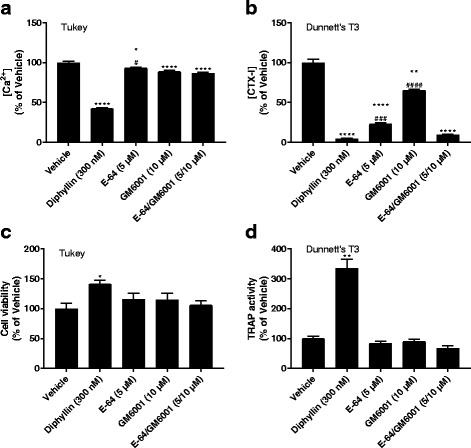
Resorption biomarkers released from osteoclasts cultured on cortical bone. Osteoclasts were cultured on bovine femoral cortical bone in the presence or absence of resorption inhibitors. Resorption of calcified extracellular matrix and collagen type I was assessed by measuring the Ca^2+^ (**a**) and C-terminal type I collagen (CTX-I) (**b**) concentrations, respectively, in the medium. Cell viability was assessed using alamarBlue (**c**) and tartrate-resistant acid phosphatase (TRAP) activity in the medium was measured for relative osteoclast quantification (**d**). Data are presented as percent of vehicle with error bars representing the SEM. Statistical significance is indicated by ^*^p < 0.05, ^**^p < 0.01, ^***^p < 0.001, ^****^p < 0.0001 for comparisons against the vehicle and ^#^*p* < 0.05, ^##^*p* < 0.01, ^###^*p* < 0.001, ^####^*p* < 0.0001 for comparisons against E-64/GM6001 (only shown for E-64 and GM6001); the post hoc test used is indicated in the top left corner of each graph

The resorption of cortical bone and the biomarkers released have been extensively described before [39]. In this study, the resorption of cortical bone in the presence of resorption inhibitors—the cysteine protease inhibitor E-64, the MMP inhibitor GM6001, and the V-ATPase inhibitor diphyllin—serve mainly as validation of functional resorption on the cortical bone slices and as a reference for the subchondral bone resorption in osteochondral ECM. For the purposes of comparing protease contribution to the different biomarker levels, the background levels were subtracted from all biomarker measurements in osteoclast-containing wells followed by normalization to the vehicle condition. Throughout this study, the cortical bone resorption parameters performed as expected (Fig. 2, Additional files 1 and 2: Figures S1 and S2). Ca^2+^ release was reduced by diphyllin (42% of vehicle, *p*_Tukey_ <0.0001) whereas protease inhibitors only had modest, albeit statistically significant, effects (Fig. 2a). Release of the bone resorption biomarker CTX-I was significantly reduced by all inhibitors (Fig. 2b), especially by diphyllin (4% of vehicle, *p*_DT3_ <0.0001) which emphasizes that the cortical bone is heavily calcified. Diphyllin increased the viability (Fig. 2c) and TRAP activity (Fig. 2d) of the cultures, as expected based on previous research [41]. C2M—a metabolite of MMP-mediated collagen type II degradation—release was not detectable above background levels (Additional file 7: Figure S7) indicating that collagen type II was not present in the matrix and was not being resorbed, nor was there any detectable cross-reactivity with resorbed collagen type I.

### Osteoclast effects on the articular cartilage compartment

**Fig. 3:**
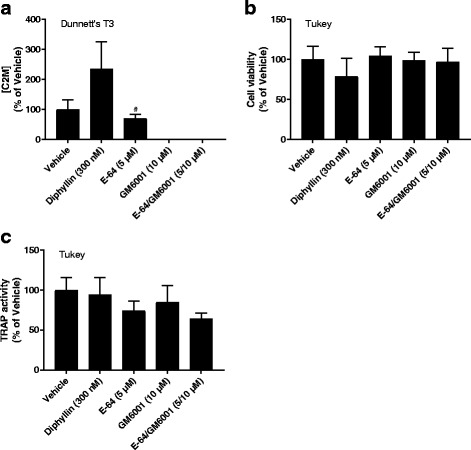
Resorption biomarkers released from osteoclasts cultured on articular cartilage. Osteoclasts were cultured on articular cartilage from bovine femoral condyles in the presence or absence of resorption inhibitors. Resorption of collagen type II was assessed by measuring C2M concentrations in the medium (**a**). Cell viability was assessed using alamarBlue (**b**) and tartrate-resistant acid phosphatase (TRAP) activity in the medium was measured for relative osteoclast quantification (**c**). Data are presented as percent of vehicle with error bars representing the SEM. Statistical significance is indicated by ^*^p < 0.05, ^**^p < 0.01, ^***^p < 0.001, ^****^p < 0.0001 for comparisons against the vehicle and ^#^*p* < 0.05, ^##^*p* < 0.01, ^###^*p* < 0.001, ^####^*p* < 0.0001 for comparisons against E-64/GM6001 (only shown for E-64 and GM6001), the post hoc test used is indicated in the top left corner of each graph

Cartilage degradation by osteoclasts was assessed by the release of C2M. There was a trend towards GM6001 completely abrogating the C2M release (0% of vehicle, *p*_DT3_ = 0.16) and E-64 reducing it to a lesser extent (69% of vehicle, *p*_DT3_ = 0.98), although neither was statistically significant (Fig. 3a). The effects of E-64 varied between trials (Additional files 3 and 4: Figures S3A and S4A). The diphyllin condition showed a trend towards increased C2M release (236% of vehicle, *p*_DT3_ = 0.79), although some data points were outside the general pattern and possibly skewed the results (Fig. 3a). This effect also varied between trials (Additional files 3 and 4: Figures S3A and S4A). There were no statistically significant effects of any inhibitor on culture viability (Fig. 3b) or osteoclast numbers (Fig. 3c). CTX-I release was not detectable above background levels (Additional file 7: Figure S7) indicating that collagen type I was not present in the matrix and was not being resorbed, nor was there any detectable cross-reactivity with resorbed collagen type II. Similarly, Ca^2+^ release was not detectable above background levels (Additional file 7: Figure S7) indicating that the cartilage was not calcified. ^342^FFGV-G2, a metabolite of MMP-mediated aggrecan degradation [42], and ^374^ARGS-G2, a metabolite of aggrecanase-mediated aggrecan degradation [42], were also measured but neither neo-epitope could be detected (data not shown).

### Osteoclast effects on the osteochondral junction compartment

**Fig. 4:**
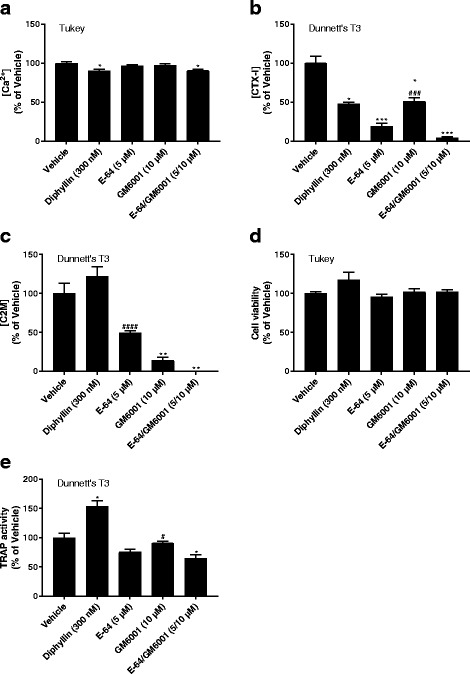
Resorption biomarkers released from osteoclasts cultured on osteochondral extracellular matrix (ECM). Osteoclasts were cultured on osteochondral ECM from bovine femoral condyles in the presence or absence of resorption inhibitors. Resorption of calcified ECM and collagen type I was assessed by measuring the Ca^2+^ (**a**) and C-terminal type I collagen (CTX-I) (**b**) concentrations, respectively, in the medium. Resorption of collagen type II was assessed by measuring C2M (**c**) concentrations in the medium. Cell viability was assessed using alamarBlue (**d**) and tartrate-resistant acid phosphatase (TRAP) activity in the medium was measured for relative osteoclast quantification (**e**). Data are presented as percent of vehicle with error bars representing the SEM. Statistical significance is indicated by ^*^p < 0.05, ^**^p < 0.01, ^***^p < 0.001, ^****^p < 0.0001 for comparisons against the vehicle and ^#^*p* < 0.05, ^##^*p* < 0.01, ^###^*p* < 0.001, ^####^*p* < 0.0001 for comparisons against E-64/GM6001 (only shown for E-64 and GM6001); the post hoc test used is indicated in the top left corner of each graph

In the osteochondral ECM we obtained resorption of both bone and cartilage components. Interestingly, the release of CTX-I (Fig. 4b) was significantly inhibited in the presence of diphyllin (50% of vehicle, *p*_DT3_ = 0.014), E-64 (19% of vehicle, *p*_DT3_ = 0.0008), GM6001 (51% of vehicle, *p*_DT3_ = 0.013), and E-64/GM6001 (4% of vehicle, *p*_DT3_ = 0.0007) whereas Ca^2+^ release was not reduced by any inhibitor (Fig. 4a), despite using well-validated dosages of the inhibitors [39]. Although there was a trend towards E-64/GM6001 reducing CTX-I more than E-64 alone (Fig. 4b) it was not statistically significant (*p*_DT3_ = 0.07); E-64/GM6001 was, however, more potent than GM6001 alone (*p*_DT3_ = 0.007). C2M release (Fig. 4c) was reduced by GM6001 (14% of vehicle, *p*_DT3_ = 0.006) and to a lesser extent by E-64, which was not statistically significant (49% of vehicle, *p*_DT3_ = 0.07), with total abrogation of C2M release when combined (0% of vehicle, *p*_DT3_ = 0.004). E-64/GM6001 was significantly different from E-64 alone (*p*_DT3_ <0.0001) but not from GM6001 (*p*_DT3_ = 0.07). The effects of E-64 alone on C2M in other trials were either smaller (78% of vehicle, Additional file 5: Figure S5C) or not present (128% of vehicle, Additional file 6: Figure S6C); neither was significant (*p*_DT3_ = 0.98 and *p*_Tukey_ = 0.37, respectively). Diphyllin had a trend towards a small increase in C2M levels (Fig. 4c) but this was non-significant (122% of vehicle, *p*_DT3_ = 0.90) and was not present in other trials (Additional files 5 and 6: Figures S5C and S6C). There were no detectable effects on cell viability by any inhibitor (Fig. 4d). The TRAP activity (Fig. 4e) increased in the diphyllin condition on osteochondral ECM (154% of vehicle, *p*_DT3_ = 0.013). While E-64 and GM6001 did not affect TRAP activity significantly (76% of vehicle, *p*_DT3_ = 0.18 and 91% of vehicle, *p*_DT3_ = 0.94), E-64/GM6001 reduced it to 65% of vehicle (*p*_DT3_ = 0.04). Similar, but non-significant, trends were seen on cortical bone (Fig. 2d) and articular cartilage (Fig. 3c). ^342^FFGV-G2 and ^374^ARGS-G2 were also measured but neither neo-epitope was detected (data not shown).

## Discussion

Inhibition of osteoclast function using bisphosphonates has underscored a potential role of targeting osteoclasts as a treatment for OA [43]. However, how osteoclasts directly affect different joint compartments in OA, except through resorption of bone, is not clear. So far clinical OA trials, in general and those inhibiting bone resorption, have failed due to lack of structural efficacy [43]. Novel approaches are needed to determine the contribution of osteoclast-derived enzymes and acidification in degrading ECMs other than bone to improve osteoclast-related structural efficacy in OA treatments. In the light of this, we conducted a study focused on osteoclast resorption mechanisms, ECM interactions and the development of a novel tool for early drug and biomarker validation.

**Fig. 5:**
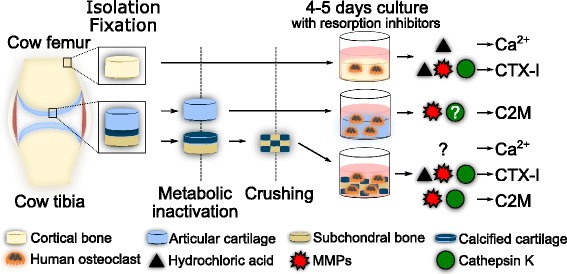
Overview of the experimental model design and results. Cortical bone, articular cartilage and an osteochondral matrix, consisting of subchondral bone and calcified cartilage, were isolated from bovine femoral diaphyses and femoral condyles, and fixated in 70% ethanol. The articular cartilage and the osteochondral matrix were metabolically inactivated by immersion in liquid nitrogen. The osteochondral matrix was crushed to generate a homogeneous osteochondral extracellular matrix (ECM) for culture. Human osteoclasts derived from CD14^+^ monocytes were cultured on the different matrices in the presence of vacuolar-type H^+^-ATPase, matrix metalloproteinase (MMP) and cathepsin K inhibitors. The contributions of hydrochloric acid, MMPs and cathepsin K in degrading the ECMs were assessed by measuring biomarkers of ECM degradation in the culture supernatants. CTX-I, C-terminal type I collagen

To our knowledge no studies have previously quantified the processes degrading articular cartilage and osteochondral junction joint compartments in a setting where osteoclasts are the known and only source of degradation. In this study, we demonstrated how osteoclasts are involved in degrading these joint compartments and characterized these processes using a combination of resorption inhibitors and tissue-specific biomarkers. In brief, our study supports osteoclast-derived cathepsin K-mediated and MMP-mediated resorption of calcified cartilage and articular cartilage independently of acidification, as measured by the C2M neo-epitope. A graphical overview of the experimental model design and the osteoclast-derived resorption processes that contributed to the ECM degradation biomarkers measured in our study can be found in Fig. 5. Using this novel cell model, we were able to investigate the role of osteoclast-derived enzymes and acidification in directly degrading articular cartilage and osteochondral junction compartments.

Osteoclasts generated inhibitable Ca^2+^ release from cortical bone but not from the osteochondral ECM—the V-ATPase inhibitor diphyllin had no effect on osteochondral Ca^2+^ release—which suggests that the osteochondral Ca^2+^ was not derived from resorption. Due to this discrepancy, reference biomarker data from cortical bone are necessary for this cell culture model to verify normal resorption and validate each experiment. As Ca^2+^ was present at equal levels in all osteochondral osteoclast conditions, i.e. Ca^2+^ levels were increased by an equal amount in all osteoclast conditions compared to background levels, we speculate that the Ca^2+^ was derived from cellular metabolism and that the osteochondral ECM is not highly calcified like cortical bone, which appears to be supported by the von Kossa staining.

Inhibiting Ca^2+^ release by targeting the V-ATPase typically raises the viability and the TRAP activity of osteoclast cultures [41, 44], likely due to Ca^2+^ release from resorption inducing osteoclast apoptosis [45]. Accordingly, TRAP activity was raised by diphyllin to a lesser extent in the osteochondral cultures than it was in the bone cultures, and similarly viability was only marginally and non-significantly raised by diphyllin in the osteochondral cultures, further supporting that the calcification of the osteochondral ECM was relatively low.

The osteochondral CTX-I levels also support the hypothesis of low calcification as CTX-I was only moderately affected by diphyllin when compared to the effects of diphyllin on cortical bone. The apparent discrepancy between the CTX-I and calcium analyses on osteochondral ECM could however be a matter of different sensitivity between the two analyses. Osteoclasts generated CTX-I from osteochondral ECM primarily through cathepsin K-mediated processes as indicated by the inhibitor panel, but secondarily also through MMP-mediated processes, in a manner similar to osteoclasts on cortical bone. Interestingly, the MMP inhibition appeared to have a stronger effect on osteochondral CTX-I release than on cortical bone CTX-I release. Similarly, osteoclasts cultured on decalcified bone display a stronger reduction in CTX-I release when MMPs are inhibited than they do on non-decalcified cortical bone, whereas the effects of cathepsin K inhibition are blunted on decalcified cortical bone, supporting that MMPs are the predominant proteases on a non-calcified matrix [8].

Osteoclast-mediated cartilage degradation has previously been demonstrated by measuring the release of glycosaminoglycans (GAGs) from osteoclasts cultured on articular cartilage [23]. In our study we instead demonstrated osteoclast-derived cartilage degradation using the C2M biomarker. C2M has previously been shown to be able to distinguish between healthy subjects and patients or between subsets of patients with arthritic diseases—such as OA [40, 46], RA [47–49] and ankylosing spondylitis [50, 51]—when measured in patient serum. Additionally, C2M has been characterized in ex vivo models of inflammatory cartilage degradation where it was shown that MMP inhibition reduces the C2M release [40]. These studies make C2M a highly relevant biomarker for both clinical studies of OA and for investigations of cartilage degradation mechanisms. Using our in vitro model we have explored additional biological mechanisms through which the C2M biomarker can be generated. Our study highlights how our model can be used for biomarker discovery and validation and for drug efficacy testing in diseases where osteoclasts may contribute to the disease process. However, it is possible that the mechanisms generating C2M in vivo and their overall contribution to C2M levels and disease may differ from the in vitro conditions.

The cartilage was degraded by osteoclast-derived MMPs in our study, as the C2M neo-epitope is mainly generated through MMP-cleavage of collagen type II and GM6001 potently inhibited the C2M release. From the osteochondral ECM we detected C2M release, which could be inhibited by E-64 and GM6001. In two of the three trials there was a trend towards cathepsin K-mediated processes contributing to C2M release from osteochondral ECM. The effect of E-64 was more apparent when it was used in combination with GM6001. This could indicate that cathepsin K contributes to release or access to the neo-epitope rather than to the generation of the neo-epitope. Cathepsin K-mediated bone resorption could also affect osteochondral C2M by enabling access to cartilage encapsulated in bone; this in turn could lead to variation in the efficacy of E-64 based on the bone-to-cartilage ratio in the matrix. Cathepsin K appeared to contribute to C2M release also on articular cartilage in two of three trials, although E-64 did not have a statistically significant effect on cartilage C2M release in any trial. The small effects of E-64 on cartilage degradation could be due to the low levels of calcification. E-64 has been shown to reduce CTX-I release from decalcified bone less than from normally calcified bone [8]. ECMs with low calcification presumably result in less acidification, thereby reducing the activity of cathepsin K as it is most effective at an acidic pH [52]. Another factor is the biomarker itself as C2M is primarily generated by MMPs. In previous studies using a model of inflammatory cartilage degradation, E-64 had no effect on C2M release [40] even though cathepsin K is expressed in this model [29]. In our study diphyllin had no inhibitory effect on cartilage C2M or osteochondral C2M, supporting the hypothesis that both non-calcified and calcified cartilage can be resorbed independently of acidification. This is in line with the findings of Touaitahuata et al. that *Dock5*^*−/−*^ murine osteoclasts degrade hypertrophic cartilage without acidifying the ECM due to dysfunctional sealing zones [53].

We were unable to detect the aggrecan degradation biomarkers ^342^FFGV-G2 and ^374^ARGS-G2 from any matrix. Although we demonstrated MMP-mediated collagen type II degradation, the MMPs present might not generate ^342^FFGV-G2 or detectable amounts thereof, possibly due to the osteoclast source and substrate affecting which MMPs are secreted [54]. It is also possible that our matrices do not contain enough aggrecan to generate detectable levels of ^342^FFGV-G2 or ^374^ARGS-G2. In a model of inflammatory cartilage degradation the ^374^ARGS-G2 neo-epitope is mainly generated by a disintegrin and metalloproteinase with thrombospondin motifs (ADAMTS)-4 [55], although ADAMTS-5 is also known to degrade aggrecan [56], but it is not known if osteoclasts express ADAMTS-4 or 5, hence, osteoclasts might not be able to generate ^374^ARGS-G2.

While a large body of evidence supports both MMPs [6, 8, 29, 30] and cathepsin K [3, 6, 8, 31] contributing to bone and cartilage degradation, their contribution in degrading calcified cartilage or in degrading cartilage in a setting where they are known to be derived from osteoclasts has to our knowledge not been demonstrated previously. To date osteoclast inhibitors have yielded mixed results in osteoarthritis clinical trials despite promise in vivo. Hence, there is a need for elucidating the contribution of different osteoclast-derived proteases to ECM degradation. In addition to demonstrating how cysteine proteases and MMPs derived from human osteoclasts may degrade knee joint ECMs, in this study we also describe how these processes can be investigated using biomarkers directly associated with the enzymatic processing of the ECM. The in vitro model itself and the novel use of biomarkers like C2M in assessing cartilage resorption is also an important finding, in addition to demonstrating calcified cartilage resorption by MMPs and cathepsins in an acidification-independent manner. Our novel model holds potential for further studies in the early development of novel drugs and biomarkers focused on knee joint ECM remodeling.

Previous studies have suggested that the ECM is an important regulator of the osteoclast phenotype [28]. For the purposes of our study it is unclear if the relative importance of the osteoclast processes required for generating a particular biomarker was related to ECM-dependent phenotype shifts or different osteoclast subtypes. Our data do however demonstrate that osteoclast functionality is highly dependent on what matrix they are resorbing, as previously suggested [8, 28, 39, 57], since the different matrices required different osteoclast proteases and amounts of acidification for degradation. A previous study showed that monocytes from patients with OA display increased osteoclastogenesis accompanied by increased bone resorption and reduced apoptosis in vitro [22]. These findings suggest a phenotype shift in OA osteoclasts, thus it may be of interest to investigate if these osteoclasts also display increased cartilage or calcified cartilage resorption with an altered biomarker profile in future studies using our model.

Our cell culture model comes with some limitations. The amount of matrix obtained from a single knee and the large number of osteoclasts required to perform the cultures with a suitable number of technical replicates limits the number of conditions that can be used, resulting in relatively low statistical power that is sensitive to variation within conditions. Increased robustness could be achieved by investigating only the matrix and compound of interest in addition to reference matrices (cortical bone and/or articular cartilage) and reference inhibitors (diphyllin, E-64, and GM6001); this was, however, not suitable for the profiling approach of our study. Some experimental outputs varied between experiments, as can be seen by comparing the data in this article (Figs. 2, 3 and 4) with the supplementary data (Additional files 1, 2, 3, 4, 5 and 6: Figures S1–S6). This could potentially be explained by differences in osteoclast quality between donors and variations in matrix content between the knee joints, further necessitating strict use of reference matrices and inhibitors to validate each experiment. Finally, due to the in vitro design of our study, the findings cannot be directly extrapolated to the OA disease process.

In our study we decided to use a molecular in vitro approach to investigate the role of osteoclasts in degrading the knee joint ECMs rather than an in vivo approach. Considering that the contribution of osteoclasts or the different ECMs to the biomarker release cannot be precisely determined in vivo, an in vitro study provides a clearer picture of the osteoclast-derived mechanisms involved in resorbing the different ECMs. Additionally, the inter-species phenotype differences observed between human and murine osteoclasts with deficits in e.g. cathepsins (or with inhibited cathepsins) [58–60] indicate that the use of non-human osteoclasts are unlikely to be an accurate representation of the human situation. The use of bisphosphonates in clinical OA trials have yielded mixed results, despite great promise in vivo, and therefore investigations of how human osteoclasts may contribute to the degradation of knee joint ECMs is of importance—something which, to our knowledge, has not been previously investigated in articular cartilage and osteochondral junction joint compartments.

## Conclusions

In conclusion, in this study we have developed a novel cell culture model to demonstrate the osteoclasts’ capability of degrading the osteochondral junction and articular cartilage joint compartments in a manner that can be assessed using tissue-specific and protease-specific biomarkers. Our study supports that osteoclasts can resorb cartilage and calcified cartilage independently of acidification. We demonstrated both MMP-mediated and cysteine protease-mediated resorption of calcified cartilage. The osteoclast functionality was highly dependent on the resorbed substrate, as different ECMs required different osteoclast processes for degradation. Our novel culture system has potential to facilitate drug and biomarker development for rheumatic diseases, e.g. osteoarthritis, with pathological osteoclast processes in specific joint compartments.

## Additional files

Additional file 1: **Figure S1.** Resorption biomarkers released from osteoclasts cultured on cortical bone in an additional trial. Osteoclasts were cultured on bovine femoral cortical bone in the presence or absence of resorption inhibitors. Resorption of calcified ECM and collagen type I was assessed by measuring the Ca^2+^ (A) and CTX-I (B) concentrations, respectively, in the medium. Cell viability was assessed using alamarBlue (C) and TRAP activity in the medium was measured for relative osteoclast quantification (D). Data are presented as percent of vehicle with error bars representing the SEM. Statistical significance is indicated by ^*^p < 0.05, ^**^p < 0.01, ^***^p < 0.001, ^****^p < 0.0001 for comparisons against the vehicle and ^#^*p* < 0.05, ^##^*p* < 0.01, ^###^*p* < 0.001, ^####^*p* < 0.0001 for comparisons against E-64/GM6001 (only shown for E-64 and GM6001); the post hoc test used is indicated in the top left corner of each graph. (PDF 32 kb)

Additional file 2: **Figure S2.** Resorption biomarkers released from osteoclasts cultured on cortical bone in pilot trial. Osteoclasts were cultured on bovine femoral cortical bone in the presence or absence of resorption inhibitors. Resorption of calcified ECM and collagen type I was assessed by measuring the Ca^2+^ (A) and CTX-I (B) concentrations, respectively, in the medium. TRAP activity in the medium was measured for relative osteoclast quantification (C). Data are presented as percent of vehicle with error bars representing the SEM. Statistical significance is indicated by ^*^p < 0.05, ^**^p < 0.01, ^***^p < 0.001, ^****^p < 0.0001 for comparisons against the vehicle; the post hoc test used is indicated in the top left corner of each graph. (PDF 28 kb)

Additional file 3: **Figure S3.** Resorption biomarkers released from osteoclasts cultured on articular cartilage in an additional trial. Osteoclasts were cultured on articular cartilage from bovine femoral condyles in the presence or absence of resorption inhibitors. Resorption of collagen type II was assessed by measuring C2M (A) concentrations in the medium. Cell viability was assessed using alamarBlue (B) and TRAP activity in the medium was measured for relative osteoclast quantification (C). Data are presented as percent of vehicle with error bars representing the SEM. Statistical significance is indicated by ^*^p < 0.05, ^**^p < 0.01, ^***^p < 0.001, ^****^p < 0.0001 for comparisons against the vehicle and ^#^*p* < 0.05, ^##^*p* < 0.01, ^###^*p* < 0.001, ^####^*p* < 0.0001 for comparisons against E-64/GM6001 (only shown for E-64 and GM6001); the post hoc test used is indicated in the top left corner of each graph. (PDF 30 kb)

Additional file 4: **Figure S4.** Resorption biomarkers released from osteoclasts cultured on articular cartilage in the pilot trial. Osteoclasts were cultured on articular cartilage from bovine femoral condyles in the presence or absence of resorption inhibitors. Resorption of collagen type II was assessed by measuring C2M (A) concentrations in the medium. TRAP activity in the medium was measured for relative osteoclast quantification (B). Data are presented as percent of vehicle with error bars representing the SEM. Statistical significance is indicated by ^*^p < 0.05, ^**^p < 0.01, ^***^p < 0.001, ^****^p < 0.0001 for comparisons against the vehicle; the post hoc test used is indicated in the top left corner of each graph. (PDF 27 kb)

Additional file 5: **Figure S5.** Resorption biomarkers released from osteoclasts cultured on osteochondral ECM in an additional trial. Osteoclasts were cultured on osteochondral ECM from bovine femoral condyles in the presence or absence of resorption inhibitors. Resorption of calcified ECM and collagen type I was assessed by measuring the Ca^2+^ (A) and CTX-I (B) concentrations, respectively, in the medium. Resorption of collagen type II was assessed by measuring C2M (C) concentrations in the medium. Cell viability was assessed using alamarBlue (D) and TRAP activity in the medium was measured for relative osteoclast quantification (E). Data are presented as percent of vehicle with error bars representing the SEM. Statistical significance is indicated by ^*^p < 0.05, ^**^p < 0.01, ^***^p < 0.001, ^****^p < 0.0001 for comparisons against the vehicle and ^#^*p* < 0.05, ^##^*p* < 0.01, ^###^*p* < 0.001, ^####^*p* < 0.0001 for comparisons against E-64/GM6001 (only shown for E-64 and GM6001); the post hoc test used is indicated in the top left corner of each graph. (PDF 33 kb)

Additional file 6: **Figure S6.** Resorption biomarkers released from osteoclasts cultured on osteochondral ECM in the pilot trial. Osteoclasts were cultured on osteochondral ECM from bovine femoral condyles in the presence or absence of resorption inhibitors. Resorption of calcified ECM and collagen type I was assessed by measuring the Ca^2+^ (A) and CTX-I (B) concentrations, respectively, in the medium. TRAP activity data could not be generated in these samples. Resorption of collagen type II was assessed by measuring C2M (C) concentrations in the medium. Data are presented as percent of vehicle with error bars representing the SEM. Statistical significance is indicated by ^*^p < 0.05, ^**^p < 0.01, ^***^p < 0.001, ^****^p < 0.0001 for comparisons against the vehicle; the post hoc test used is indicated in the top left corner of each graph. (PDF 27 kb)

Additional file 7: **Figure S7.** Osteoclast-derived biomarker levels compared to background levels. Biomarkers were measured in medium from wells containing only matrix (Background, white) or matrix and osteoclasts (Osteoclasts, black), cultured on bovine femoral cortical bone (Bone), articular cartilage (Cartilage) or osteochondral ECM (Osteoch.). Resorption of calcified ECM and collagen type I was assessed by measuring the Ca^2+^ (A) and CTX-I (B) concentrations, respectively, in the medium. Resorption of collagen type II was assessed by measuring C2M (C) concentrations in the medium. TRAP activity in the medium was measured for relative osteoclast quantification (D). Data from one representative resorption assay are presented as the mean of the measured parameters in background wells or osteoclast-containing wells. Error bars represent the SEM. (PDF 32 kb)

## Abbreviations

ADAMTS: A disintegrin and metalloproteinase with thrombospondin motifs
ANOVA: Analysis of variance
Ca^2+^: Calcium
CPC: *o*-Cresolphthalein complexone
CTX-I: C-terminal type I collagen fragments
DMSO: Dimethyl sulfoxide
DT3: Dunnett’s T3 post hoc test
ECM: Extracellular matrix
EDTA: Ethylenediaminetetraacetic acid
ELISA: Enzyme-linked immunosorbent assay
GAG: Glycosaminoglycan
M-CSF: Macrophage colony-stimulating factor
MMP: Matrix metalloproteinase
NaCl: Sodium chloride
NaOH: Sodium hydroxide
OA: Osteoarthritis
RA: Rheumatoid arthritis
RANKL: Receptor activator of nuclear factor kappa-B ligand
SEM: Standard error of the mean
TRAP: Tartrate-resistant acid phosphatase
V-ATPase: Vacuolar-type H^+^-ATPase
