# Supplementary figures and images for: Osteoclasts degrade bone and cartilage knee joint compartments through different resorption processes
Source: Arthritis Res Ther. 2018 Apr 10;20:67. doi: 10.1186/s13075-018-1564-5 (PMC5894194; doi:10.1186/s13075-018-1564-5)

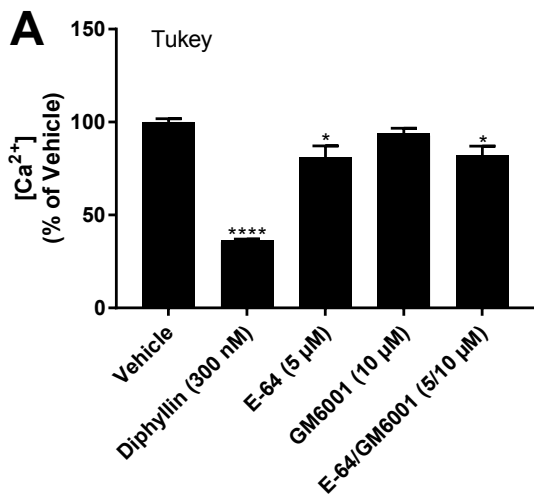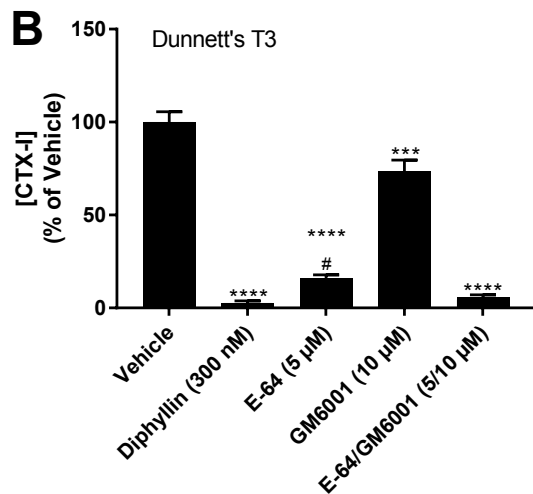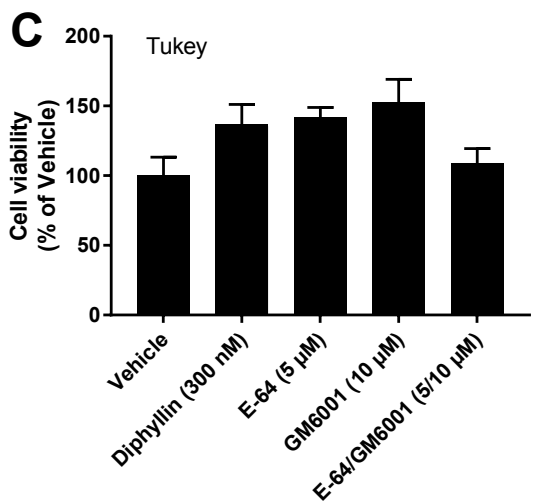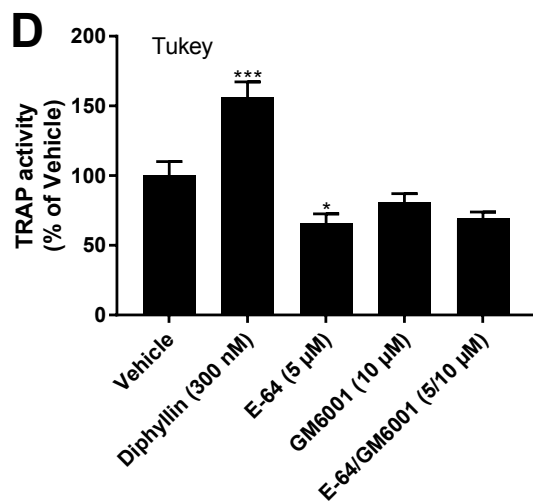

Supplement: Supplementary file 1 — Figure S1. Resorption biomarkers released from osteoclasts cultured on cortical bone in an additional trial. Osteoclasts were cultured on bovine femoral cortical bone in the presence or absence of resorption inhibitors. Resorption of calcified ECM and collagen type I was assessed by measuring the Ca2+ (A) and CTX-I (B) concentrations, respectively, in the medium. Cell viability was assessed using alamarBlue (C) and TRAP activity in the medium was measured for relative osteoclast quantification (D). Data are presented as percent of vehicle with error bars representing the SEM. Statistical significance is indicated by *p < 0.05, **p < 0.01, ***p < 0.001, ****p < 0.0001 for comparisons against the vehicle and #p < 0.05, ##p < 0.01, ###p < 0.001, ####p < 0.0001 for comparisons against E-64/GM6001 (only shown for E-64 and GM6001); the post hoc test used is indicated in the top left corner of each graph. (PDF 32 kb) [file 13075_2018_1564_MOESM1_ESM.pdf]

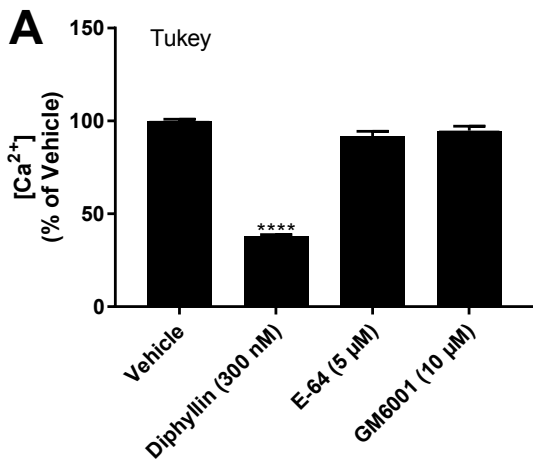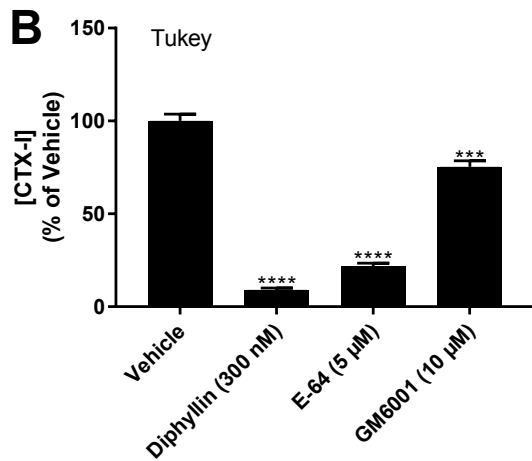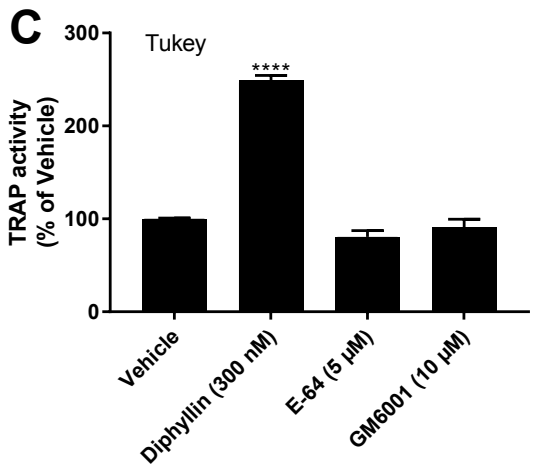

Supplement: Supplementary file 2 — Figure S2. Resorption biomarkers released from osteoclasts cultured on cortical bone in pilot trial. Osteoclasts were cultured on bovine femoral cortical bone in the presence or absence of resorption inhibitors. Resorption of calcified ECM and collagen type I was assessed by measuring the Ca2+ (A) and CTX-I (B) concentrations, respectively, in the medium. TRAP activity in the medium was measured for relative osteoclast quantification (C). Data are presented as percent of vehicle with error bars representing the SEM. Statistical significance is indicated by *p < 0.05, **p < 0.01, ***p < 0.001, ****p < 0.0001 for comparisons against the vehicle; the post hoc test used is indicated in the top left corner of each graph. (PDF 28 kb) [file 13075_2018_1564_MOESM2_ESM.pdf]

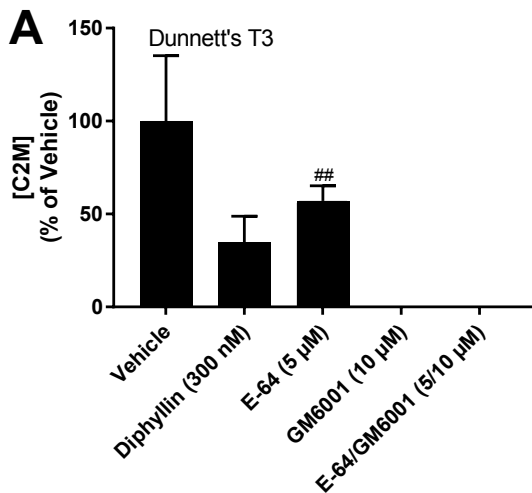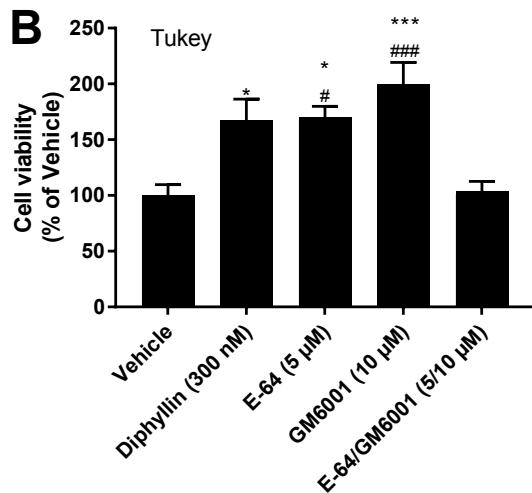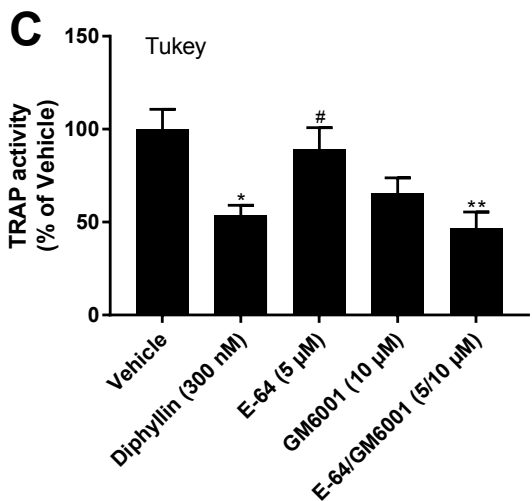

Supplement: Supplementary file 3 — Figure S3. Resorption biomarkers released from osteoclasts cultured on articular cartilage in an additional trial. Osteoclasts were cultured on articular cartilage from bovine femoral condyles in the presence or absence of resorption inhibitors. Resorption of collagen type II was assessed by measuring C2M (A) concentrations in the medium. Cell viability was assessed using alamarBlue (B) and TRAP activity in the medium was measured for relative osteoclast quantification (C). Data are presented as percent of vehicle with error bars representing the SEM. Statistical significance is indicated by *p < 0.05, **p < 0.01, ***p < 0.001, ****p < 0.0001 for comparisons against the vehicle and #p < 0.05, ##p < 0.01, ###p < 0.001, ####p < 0.0001 for comparisons against E-64/GM6001 (only shown for E-64 and GM6001); the post hoc test used is indicated in the top left corner of each graph. (PDF 30 kb) [file 13075_2018_1564_MOESM3_ESM.pdf]

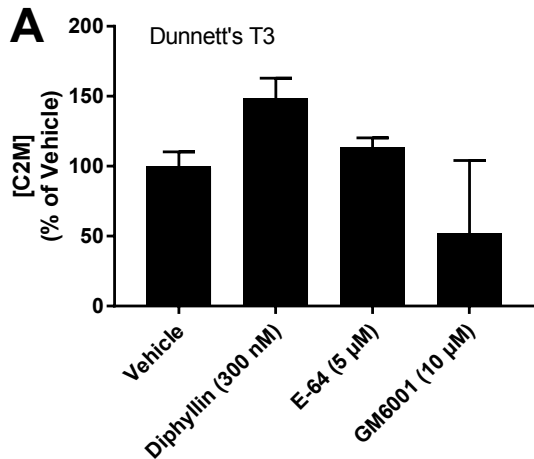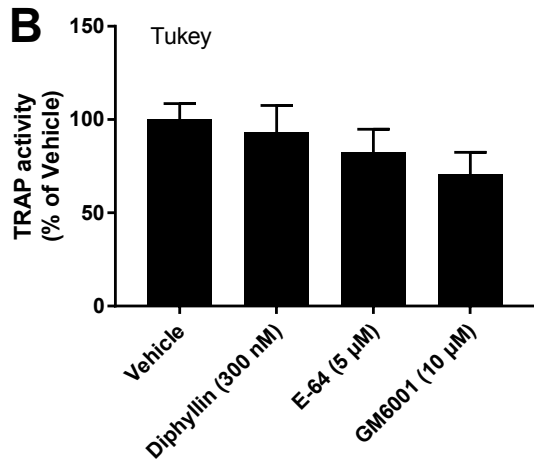

Supplement: Supplementary file 4 — Figure S4. Resorption biomarkers released from osteoclasts cultured on articular cartilage in the pilot trial. Osteoclasts were cultured on articular cartilage from bovine femoral condyles in the presence or absence of resorption inhibitors. Resorption of collagen type II was assessed by measuring C2M (A) concentrations in the medium. TRAP activity in the medium was measured for relative osteoclast quantification (B). Data are presented as percent of vehicle with error bars representing the SEM. Statistical significance is indicated by *p < 0.05, **p < 0.01, ***p < 0.001, ****p < 0.0001 for comparisons against the vehicle; the post hoc test used is indicated in the top left corner of each graph. (PDF 27 kb) [file 13075_2018_1564_MOESM4_ESM.pdf]

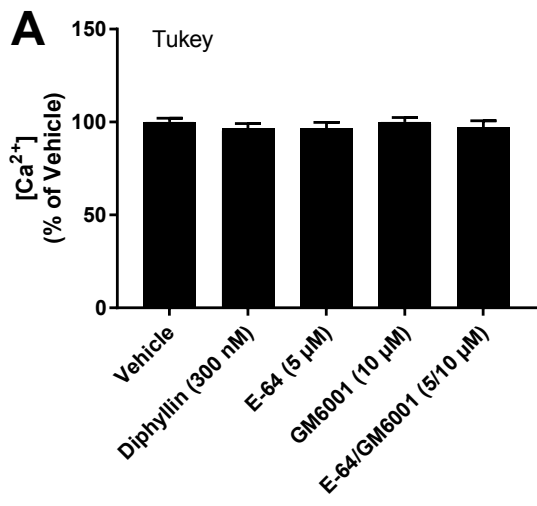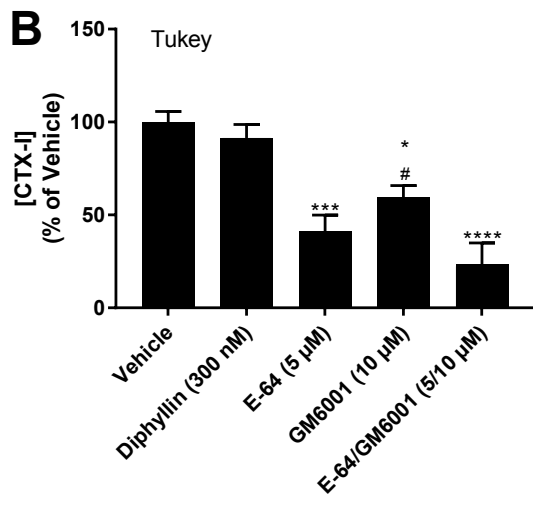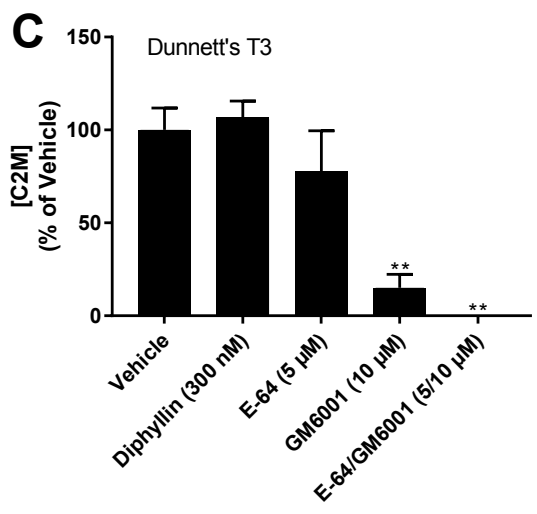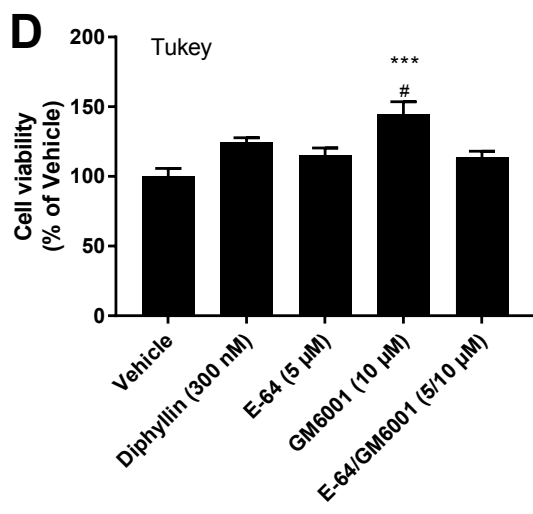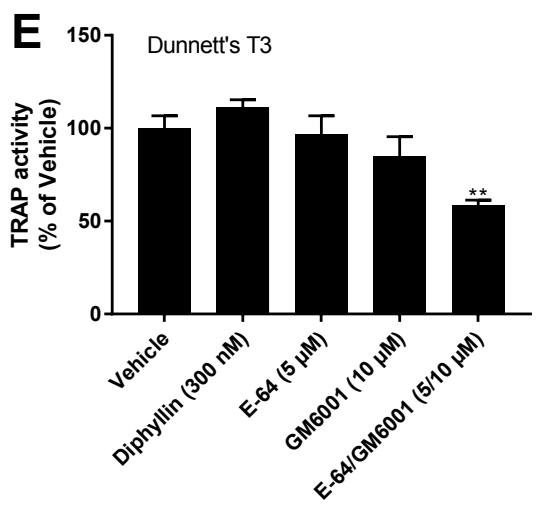

Supplement: Supplementary file 5 — Figure S5. Resorption biomarkers released from osteoclasts cultured on osteochondral ECM in an additional trial. Osteoclasts were cultured on osteochondral ECM from bovine femoral condyles in the presence or absence of resorption inhibitors. Resorption of calcified ECM and collagen type I was assessed by measuring the Ca2+ (A) and CTX-I (B) concentrations, respectively, in the medium. Resorption of collagen type II was assessed by measuring C2M (C) concentrations in the medium. Cell viability was assessed using alamarBlue (D) and TRAP activity in the medium was measured for relative osteoclast quantification (E). Data are presented as percent of vehicle with error bars representing the SEM. Statistical significance is indicated by *p < 0.05, **p < 0.01, ***p < 0.001, ****p < 0.0001 for comparisons against the vehicle and #p < 0.05, ##p < 0.01, ###p < 0.001, ####p < 0.0001 for comparisons against E-64/GM6001 (only shown for E-64 and GM6001); the post hoc test used is indicated in the top left corner of each graph. (PDF 33 kb) [file 13075_2018_1564_MOESM5_ESM.pdf]

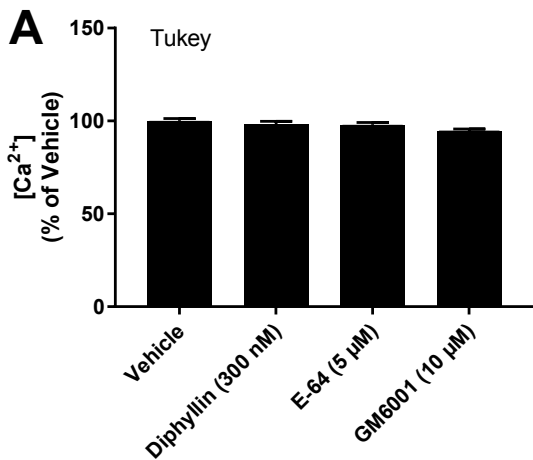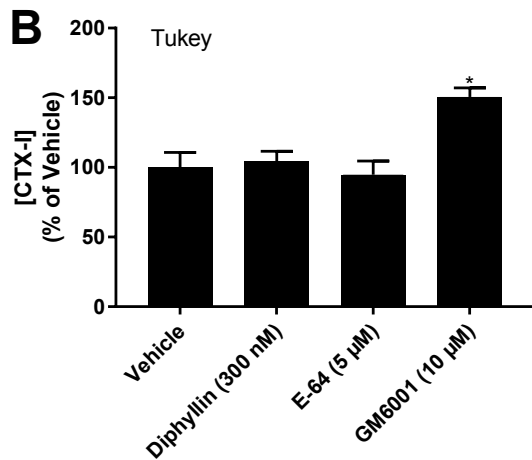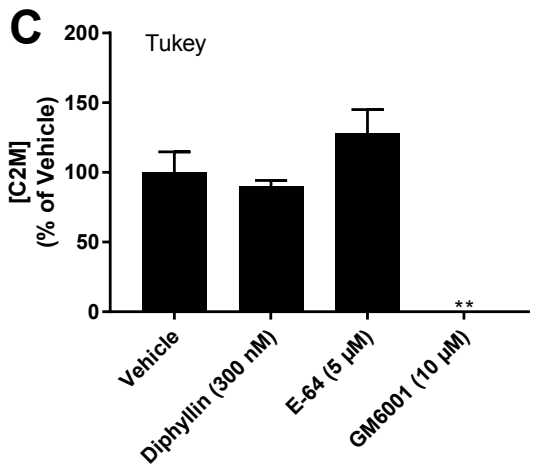

Supplement: Supplementary file 6 — Figure S6. Resorption biomarkers released from osteoclasts cultured on osteochondral ECM in the pilot trial. Osteoclasts were cultured on osteochondral ECM from bovine femoral condyles in the presence or absence of resorption inhibitors. Resorption of calcified ECM and collagen type I was assessed by measuring the Ca2+ (A) and CTX-I (B) concentrations, respectively, in the medium. TRAP activity data could not be generated in these samples. Resorption of collagen type II was assessed by measuring C2M (C) concentrations in the medium. Data are presented as percent of vehicle with error bars representing the SEM. Statistical significance is indicated by *p < 0.05, **p < 0.01, ***p < 0.001, ****p < 0.0001 for comparisons against the vehicle; the post hoc test used is indicated in the top left corner of each graph. (PDF 27 kb) [file 13075_2018_1564_MOESM6_ESM.pdf]

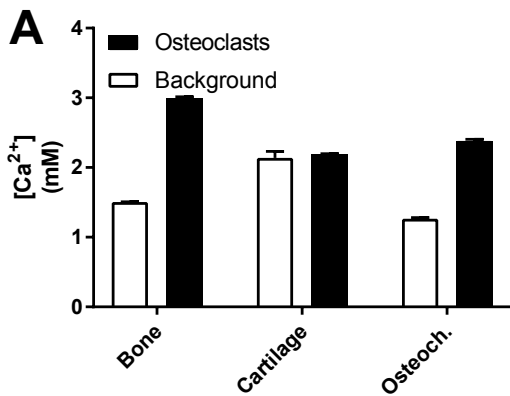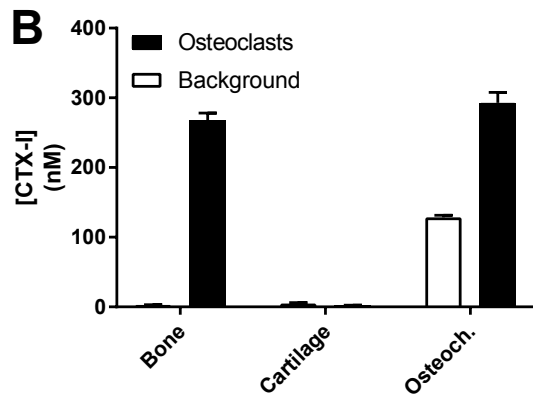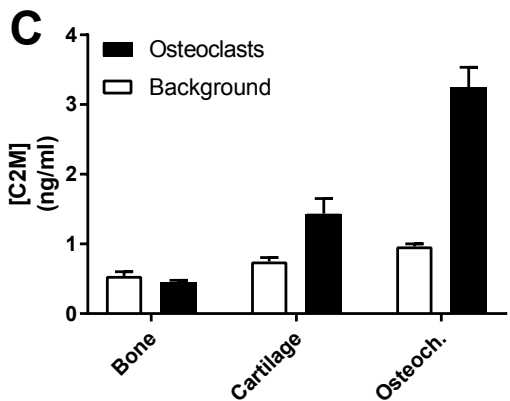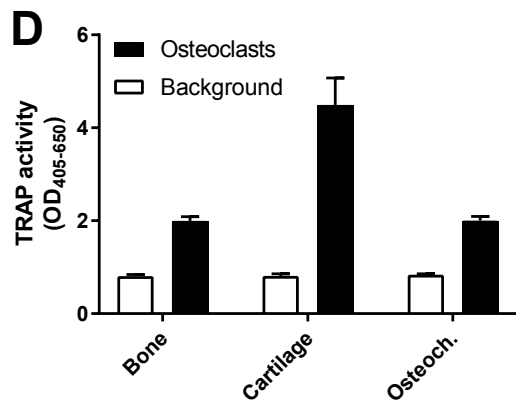

Supplement: Supplementary file 7 — Figure S7. Osteoclast-derived biomarker levels compared to background levels. Biomarkers were measured in medium from wells containing only matrix (Background, white) or matrix and osteoclasts (Osteoclasts, black), cultured on bovine femoral cortical bone (Bone), articular cartilage (Cartilage) or osteochondral ECM (Osteoch.). Resorption of calcified ECM and collagen type I was assessed by measuring the Ca2+ (A) and CTX-I (B) concentrations, respectively, in the medium. Resorption of collagen type II was assessed by measuring C2M (C) concentrations in the medium. TRAP activity in the medium was measured for relative osteoclast quantification (D). Data from one representative resorption assay are presented as the mean of the measured parameters in background wells or osteoclast-containing wells. Error bars represent the SEM. (PDF 32 kb) [file 13075_2018_1564_MOESM7_ESM.pdf]
